# Performance-Based Executive Function Instruments Used by Occupational Therapists for Children: A Systematic Review of Measurement Properties

**DOI:** 10.1155/2021/6008442

**Published:** 2021-08-06

**Authors:** Ivan Neil B. Gomez, Sharleen Alyssa M. Palomo, Ana Melissa U. Vicuña, Jose Antonio D. Bustamante, Jillian Marie E. Eborde, Krishna A. Regala, Gwyn Marie M. Ruiz, Andrea Lorraine G. Sanchez

**Affiliations:** ^1^Center for Health Research and Movement Science, University of Santo Tomas, Manila, Philippines; ^2^Department of Occupational Therapy, College of Rehabilitation Sciences, University of Santo Tomas, Manila, Philippines; ^3^The Graduate School, University of Santo Tomas, Manila, Philippines

## Abstract

**Introduction:**

The use of executive function (EF) instruments to assess children's functional performance is obscured with a lack of consensus on which is most suitable to use within the occupational therapy profession. This review identifies EF instruments used by occupational therapists (OTs) for children and evaluates their measurement properties.

**Methods:**

This systematic review was registered in PROSPERO (CRD42020172107). We reviewed occupational therapy-related studies published until March 2021, to identify performance-based EF instruments used among children by OTs. Two review authors independently screened, extracted, and evaluated the methodological rigor of the included studies. Adequacy of the measurement properties was determined using the COSMIN, and the Terwee criteria were used for synthesis of best evidence.

**Results:**

Five EF assessments were found across eight study articles: Behavioural Assessment of the Dysexecutive Syndrome for Children, Children's Cooking Task, Children's Kitchen Task Assessment, Do-Eat, and Preschool Executive Task Assessment. Adequacy of measurement properties and synthesis of best evidence varied, leading to a low GRADE rating on the certainty of evidence for the included instruments.

**Conclusions:**

There is limited evidence that supports the certainty of evidence on the measurement properties of the reviewed tools in helping OTs assess performance-based EF among children. Nevertheless, the authors conditionally suggest their use based on the critical need to measure children's EF. Further research is needed to establish the measurement properties of these measures across different childhood populations.

## 1. Introduction

Executive function (EF) is an umbrella term that incorporates a collection of interrelated processes responsible for purposeful, goal-directed behaviour [1]. It encompasses various processes (i.e., cognitive flexibility, working memory, and response inhibition), which play a key role in regulating goal-oriented behaviour and can support function in children [2, 3]. EFs traditionally have been assessed through standardized psychometric measurements [3]. Standardized assessments provide health-related professionals a clinical picture of a person's ability to perform activities necessary to develop a comprehensive assessment of intervention effectiveness, comparing clinical groups, and outcome monitoring [4, 5]. Standardization entails a rigorous process of examining the psychometric properties (i.e., validity and reliability) of assessment tools [4]. However, the use of standardized assessments in allied health professions has been low due to issues related to resources (i.e., time, financial, and limited clinician's knowledge) [6, 7].

Traditionally, EF assessments have been carried out using standardized laboratory-based measurements within a controlled environment [3, 8]. Although neuropsychological measures provide good indicators of fundamental cognitive and executive components, neuropsychological tests' performance is often not predictive of real-world complex task performance and functional ability [9–11]. Daily life performance and the executive abilities that support it often require multitasking and the generation and implementation of adaptive strategies to accommodate novel environments and perform tasks in the real world [9, 11, 12]. EF measures that are used for school-aged children were originally designed and validated for adult populations. When these measures are administered, they should be scaled down towards a version applicable for children and take into consideration that children have different skills, the level of complexity of the tasks, and the developmental context. Therefore, measures with definite norms and better performance should be considered [13]. Furthermore, research on EF assessments has also been through tests that measure singular processes, instead of from a pluralistic perspective reflecting EF's fundamental construct. Assessment should contain an array of EFs necessary for complex life tasks encompassing real-world contexts [8, 9, 11].

Different allied health professionals have been reported to be responsible for the assessment and intervention related to EF. OTs are part of that allied health team concerned with determining cognitive abilities needed in everyday task performance to perform various activities [14]. These cognitive abilities fall within the EF domains, whose main outcome reflects performance in daily activities and how it contributes to functional independence [8, 11]. Critical to the occupational therapy process is a thorough and comprehensive evaluation of a child's EF that may influence their abilities to participate in childhood occupations [8]. This is enabled by using adequate measures of EF assessment tools that consider their occupations and contexts. Given the importance of EFs in children's daily activities, preference should be on performance-based assessments within naturalistic contexts [8, 15]. While there are several available EF tools for children, it is crucial that ecologically valid and performance-based assessments be used in occupational therapy. However, there is no existing systematic review that produces evidence on the measurement properties of EF tools used in children in occupational therapy. Therefore, it is imperative to review the extant evidence base that supports performance-based EF instruments used by OTs for children and examine their measurement properties.

## 2. Objectives

This systematic review is aimed at identifying performance-based EF instruments used by OTs for children and evaluating their measurement properties.

## 3. Methods

This systematic review was registered with PROSPERO (CRD42020172107) and written based on the recommendation of PRISMA [16].

### 3.1. Search Strategy and Selection Criteria

Possible articles for inclusion were searched using the following databases: PubMed, Scopus, CINAHL, EBSCO, MEDLINE, and Google Scholar. In the likelihood that some relevant articles might be missed, we also performed hand searching through known occupational therapy journals, using an initially preestablished and tested search strategy (Supplementary File 1). Two review authors searched the source systems until March 2021. No time filter was applied. A three-level selection process was used. Any disagreements between the review authors were sorted through consensus discussion or a third review author. Articles were included if they met the following criteria: reported on a performance-based EF assessment used in children up to 12 years old; developed, used, or tested by an OT in the study; the instrument measures several EF processes; must report their result of at least one measurement property conducted within the study; published in a peer-reviewed journal; and must have an English version if written in a different language.

### 3.2. Evaluation of Methodological Quality and Measurement Properties

In assessing the methodological quality, design, and reporting of the included studies, we used the COSMIN criteria (Supplementary File 2) to evaluate measurement properties' risk of bias within studies. The assessment of methodological quality was accomplished by two independent review authors, with a consensus or a third author being consulted when a conflict in rating arose.

### 3.3. Data Extraction and Synthesis

The psychometric properties of the reported EF tools in the reviewed articles were extracted using the data extraction form from the Joanna Briggs Institute, which outlines the specific constructs assessed, country/language/culture, mode of administration, setting/context, participant characteristics, results (measurement properties), and authors' comments. Information on measurement properties was based on the COSMIN taxonomy [17]. Two review authors extracted the data, with a third review author mitigating differences.

The pooled summaries of the reported EF instruments are presented in a summary table and further discussed using a narrative synthesis. To provide the best synthesis measure (Supplementary File 3), we used the levels of evidence for the overall quality of the measurement property, previously usedby Dobson et al. [18]), adapted from Terwee et al.[19]). The summarized evidence in this review was evaluated in its certainty using the Grading of Recommendations Assessment, Development and Evaluation (GRADE; Supplementary File 4). While the GRADE assessment is mainly subjective, we used the rating recommendations suggested for each criterion component to identify the quality of evidence and strength of recommendation.

## 4. Results

### 4.1. Study Selection

**Figure 1:**
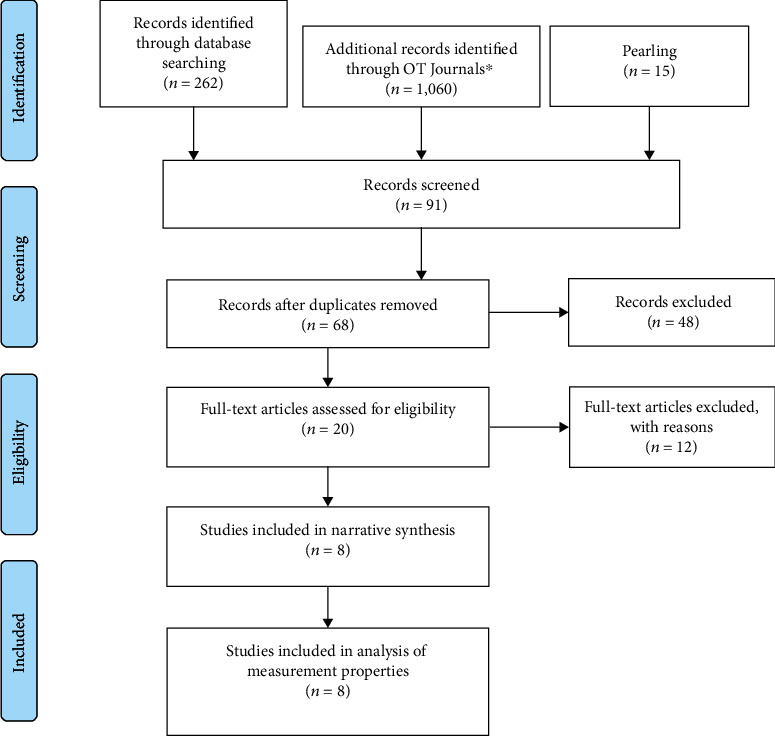
PRISMA flow diagram.

The comprehensive search resulted in 1,337 articles across all databases and sources. After the first screening level, 91 articles were filtered for duplicates, which resulted in 68 articles eligible for title and abstract screening. Full-text article screening was performed on 20 studies, with 12 articles excluded for reasons of not completely meeting the review criteria. Only eight articles were included in the final review using narrative synthesis and analysis of measurement properties. A summary of the study selection procedures is outlined in Figure 1.

### 4.2. Study Characteristics

The eight included articles reported on five performance-based EF assessment tools: (1) Behavioural Assessment of the Dysexecutive Syndrome for Children (BADS-C) [20], (2) Children's Cooking Task (CCT) [21–23], (3) Children's Kitchen Task Assessment (CKTA) [24], (4) Do-Eat [25, 26], and (5) PETA [27]. In combination, the tools were tested among typically developing children and children with conditions between the ages of 5-12 years old (*n* = 684) from different ethnic and cultural backgrounds in several countries. A summary of the extracted information from each study is presented in Table 1. The measurement properties for the EF instruments are summarized in Table 2. In assessing the adequacy of each EF tool's measurement properties as reported in the individual studies, a summary is reported in Table 3. The summary of the best evidence of each tool across studies and the GRADE rating of evidence certainty can be found in Table 4.

### 4.3. Behavioural Assessment of the Dysexecutive Syndrome for Children (BADS-C)

BADS-C [28] is a standardized assessment battery that examines EF in children and adolescents. It contains five developmentally appropriate measures and one questionnaire, amounting to six administered tests. The defined EF areas examined by the BADS-C include (1) inflexibility, (2) perseverance, (3) novel problem solving, (4) impulsivity, (5) planning, and (6) the ability to use feedback to moderate behaviour. The subtests performed were the following: (1) playing card test, (2) water test, (3) key search test, (4) zoo map test 1, (5) zoo map test 2, and (6) six-part test. Each subtest had its scoring guideline. Generally, scores were derived from the number of tasks completed correctly and any broken rules or errors committed.

We found one article that tested the psychometric property of BADS-C within the occupational therapy field [20] among Hebrew-speaking Israeli children aged 8-15 years. The instrument was translated from its original English version to Hebrew, using forward and backward translation by a bilingual clinician to ensure cross-cultural validity. The construct validity showed significant differences between the different age groups on: playing card test (*p* < 0.0001), water test (*p* = 0.001), key search test (*p* < 0.0001), and zoo map test (*p* < 0.0001). The study presented no significant correlations for gender, socioeconomic status, and parents' educational status when it came to the level of performance in children that undertook the BADS-C.

Construct validity was noted to have at least 75% of the results following the hypotheses, whereas cross-cultural validity significantly lacked information as multiple group factor analysis and DIF analysis were not performed. The content validity, structural validity, internal consistency, reliability, and criterion validity were not examined. On the other hand, strong construct validity and limited cross-cultural validity were recorded for the BADS-C. Overall, the GRADE rating certainty in the evidence for BADS-C was low due to limitations in study quality, sparse data, and probability of reporting bias.

### 4.4. Children's Cooking Task (CCT)

CCT was adapted from the Adult Cooking Task to be suitable for children [21]. CCT is a performance-based assessment that measures a child's EF while doing an open-ended real-life cooking task. The tasks include preparing a chocolate cake and a fruit cocktail while following a recipe using the necessary ingredients and utensils on a table. Three published articles assessed its measurement properties among typically developing children and children with traumatic brain injury in France [22] and Australia [21] and typically developing children and children with EF disorder in Israel [23]. The published studies did not explicitly state the EF measured; however, the execution error assessed by the tool is related to volition, planning, goal direction, and task monitoring. The scoring is based on a classification and quantification of errors and a qualitative analysis of the task.

Reliability properties were only reported in two articles. Internal consistency was found to be good at Cronbach's *α* = 0.86 [21]. Interrater reliability based the different scores in the CCT was inconsistent, with ICC ranging from poor to excellent: total number of errors: ICC = 0.96; types of errors: ICC = 0.70–0.99; and substitution-sequence errors: ICC = 0.37 [22]. A similar range of scores was found for the test-retest reliability of CCT [21]: total number of errors: ICC = 0.89; duration of the task: ICC = 0.94; types of error: ICC = 0.75 − 0.90; substitution-inversion: ICC = 0.68; estimation errors: ICC = 0.46; and purposeless action: ICC = 0.59.

Validity properties were reported in all three articles; however, these were mainly limited to construct and criterion validity. The original version of CCT was in French and translated into English [21] and Hebrew [23] to fit the cultural contexts tested. Aside from language translation, certain aspects of the items were mildly culturally modified (i.e., utensils and measurement units). Construct validity was tested for age and group differences. The total number of errors in the CCT significantly decreased with age in the control group (rs = −0.454; *p* < 0.04) and in the TBI group (rs = −0.552; *p* = 0.004) [21]. Significant group differences were found between the total number of errors between typically developing and clinical groups (*p* < 0.001) across the three studies. However, conflicting results were found for the specific error types and qualitative analysis. Criterion validation across the three studies was also varied and inconsistent using different comparative measures. One study [22] found no significant correlation between the total number of errors in the cooking task and the scores on the different neuropsychological tests or behavioural questionnaires. In another study [21], the participant's performance in the CCT was significantly correlated (*p* ≤ 0.05) to general cognitive ability and some of the cognitive tests of executive functions on the D-KEFS. The most recent study [23] reports a moderate positive correlation between the BRIEF-SR subscales plan/organization (*r* = 0.31, *p* ≤ 0.05) and task duration.

The extant evidence fails to provide a report on the content and structural validity of the CCT. There is limited evidence on its internal consistency, with only one study of fair quality due to the number of samples recruited. The overall rating for reliability was deemed limited, as the two articles that reported reliability focused on different types of reliability. While there are significant differences across age and clinical groups, the current evidence was only found for the total number of errors and not for the other measures found in the CCT. Similarly, criterion validity was inconsistent across studies, where item scores on the CCT did not correlate with the varied comparative tools used. Nevertheless, the CCT was the most frequent tool reviewed, with translations and cultural adaptations. Albeit, the specific results of the cross-cultural validation were incompletely reported or reported elsewhere. Overall, the GRADE rating for certainty in the evidence of CCT was low due to limitations in study quality, imprecise or sparse data, inconsistency, and a possible reporting bias.

### 4.5. Children's Kitchen Task Performance

Developed by American OTs, the CKTP is an iteration of the Kitchen Task Assessment (KTA; Baum & Edwards [29]) intended for adults. The CKTP involves assessing the child's EF as they are engaged in a functional and age-appropriate activity (i.e., making playdough) that simulates a simple cooking task seen in the original KTA. Only one article was found to describe the measurement properties of CKTP [24]. In reviewing the measurement properties of CKTP, [24]) were able to describe its development process and report on its reliability (interrater reliability and internal consistency) and validity (discriminant validity). The administration of CKTP is performed by an OT and is aimed at examining a child's EF skills in the areas of initiation, organization, planning and sequencing, judgment and safety, and completion. These EF processes are represented in the specific tasks within the instrument. Scoring is based on the level of cues given. The available measurement properties of CKTP were tested among African-American, English-speaking children.

Reliability properties report an internal consistency of Cronbach's *α* = 0.68 and excellent interrater reliability (ICC = 0.98). Validity testing is limited to discriminant validity, where the CKTP has been shown to detect improved EF in task performance among older participants, but this did not reach statistical significance (*F*[4, 45] = 3.83, *p* < 0.008). However, CKTP can discriminate high and low scoring children when compared to EF assessments: (1) BRIEF (inhibition: *p* < 0.003; BRI: *p* ≤ 0.01); (2) D-KEFS Confirmed Correct Card Sorts (*p* < 0.03); and (3) WISC-IV Digit Span backwards (*p* < 0.04).

The CKTP falls short in addressing evidence on content, structural, and criterion validity. While internal consistency is reported, the results do not provide concrete evidence on unidimensionality or positive structural validity, with its Cronbach's *α* falling short of the <0.70 thresholds. Construct validity, through its ability to discriminate age and performance indicators (i.e., high vs. low), was not consistent with the intended hypothesis. Nevertheless, the CKTP has shown excellent interrater reliability. Thus, while this review finds limited support on its internal consistency and construct validity in the synthesis of best evidence, there is moderate evidence on its interrater reliability. Overall, the GRADE rating certainty in the evidence for CKTA was low due to limitations in study quality, imprecise or sparse data, and inconsistency.

### 4.6. Do-Eat

Do-Eat is a set of questionnaire and test that measures a child's task performance, sensory-motor skills, and EF as they participate in three tasks: (1) make a sandwich, (2) prepare chocolate milk, and (3) fill out a certificate of outstanding performance. This review found two published articles that examined its measurement properties among 5-9-year-old Israeli typically developing children and children with DCD [25] or ADHD [26]. The test is administered by an OT and can measure EF processes of attention, initiation, sequencing, transition from one activity to another, spatial and temporal organization, inhibition, problem solving, and remembering instructions, with a scoring of 1-5 (increasing range).

In both articles, Do-Eat has high internal consistency ranging from 0.877 to 0.890. Interrater reliability was only examined among typically developing children and children with DCD [25] and ranged from 0.92 to 1.00. In the same article, content and face validation by OTs was reported, but no specific statistical data was provided. Both articles reported on construct validity (discriminant and concurrent validity), with conflicting results. Do-Eat is reported to discriminate between typically developing children and children with DCD (*t*[57] = 6.92, *p* < 0.001) by [25]) but was not correlated with any EF assessment. However, [26]) were able to find significant correlations on the Do-Eat EF task (preparing chocolate milk) and BRIEF (BRI and MI) subscales (*r* = 0.49, *p* ≤ 0.05; *r* = 0.47, *p* ≤ 0.05, respectively).

Do-Eat fails to provide evidence on its structural and criterion validity. Synthesis of best evidence considered both articles due to the limited availability of evidence. While high internal consistency is reported, there is still limited evidence for exceptionality in its unidimensionality and positive structural validity. High interrater reliability was only reported in one study. The correlation between Do-Eat's EF tasks with other established EF assessments is inconsistent. Considering this, the synthesis of best evidence on Do-Eat's measurement properties suggests limited evidence on its content validity, moderate evidence on its internal consistency and interrater reliability, and conflicting evidence on its structural validity. Overall, the GRADE rating certainty in Do-Eat's evidence was low due to limitations in study quality, uncertainty indirectness, and possible reporting bias.

### 4.7. Preschool Executive Task Assessment (PETA)

PETA was developed to measure children's EF using ecologically valid measures (Burgess et al. [30]). EF functions measured by the tool include working memory, distractibility, organization, and emotional control. The tool's measures included a scoring system independent from a person's level of functioning and ability in linguistics and motor skills; it focuses on the process of the child in doing a multistep task that could be appropriately seen in the context of the classroom. It consists of both qualitative and quantitative scoring values that cater to different dimensions of interest. We found one study that fit into our review criteria that tested PETA within a population of 166 typically developing English preschool-aged children from the UK [27].

PETA reports good to excellent reliability (interrater reliability: ICC = 0.93; intrarater reliability: ICC = 0.88 − 0.98). PETA was only tested among typically developing children ages 3-6. Age as a construct was validated, and results suggest an increase in EF with a child's age (*p* ≤ 0.001). Chronological age predicted 40% of the variance in TS (*p* ≤ 0.001). Age was also strongly related to performance on all quantitative domains of the PETA (TS, TC, initiation, sequencing, metacognition, completion, time for completion; *p* ≤ 0.005), except for judgment/safety. Criterion validity was tested between PETA and BRIEF. A significant association was observed between the PETA TS and the BRIEF-P GEC (*p* ≤ 0.001). Other correlations were not significant.

Among the reviewed studies, PETA is the most recent, which may contribute to the findings that it still fails to provide salient evidence on its content and structural validity and internal consistency. Its reliability properties were deemed strong. However, its construct validity failed to provide convincing reports of age effects across its different scores. Likewise, there is limited evidence on its criterion validity property as the correlations reported were restricted to only significant findings for one measure. Overall, the GRADE rating certainty in the evidence for PETA was noted to be low due to limitations in inconsistency and sparse and imprecise data.

## 5. Discussion

The evidence on the use of performance-based EF assessment among children in OT practice is limited. This review has highlighted a few articles discussing only five performance-based EF instruments for children: BADS-C, CCT, CKTP, Do-Eat, and PETA. The measurement properties of these EF instruments are at times lacking or conflicting, which substantiates the low certainty of evidence supporting them.

EFs are sets of higher cognitive processes that enable children the ability to participate in various age-related activities [31, 32]. While there are a number of available EF instruments available, the use of performance-based instruments is a key concept that is important in OT practice. Performance-based assessments can link EF with performance in occupations (Burgess et al. [30]). They provide ecologically valid measures of EF outcomes in an authentic context that requires multitasking and reflects the press of everyday task performances [8, 11]. To an extent, performance-based EF assessments ground themselves in an occupation-based perspective. The results in this review emphasize the need to develop EF instruments that can provide a picture of a child's participation in age-related occupations.

EF assessments in OT practice have gained attention in recent years [33, 34]. The types of EF instruments used for childhood populations include pen and paper proxy-reported measures (i.e., parent answered), laboratory-based procedures (i.e., computer software), and performance-based assessments. One of the more common traditional pen and paper EF assessment tools found in OT literature is the BRIEF [35], a parent-answered questionnaire that measures EF in individuals aged 5-18 using items formulated to reflect activities in daily life. While it has been suggested to have ecological validity [36], it lacks occupation-based contexts, which is important in OT evaluation. In the more recent years, alternative new forms of EF instruments have been suggested in the form of computerised or virtual reality testing [37]. The Jansari assessment of Executive Functions for Children (JEF-C) is a computerised EF assessment in a nonimmersive gamified virtual environment [15]. While both of these tools may seem ecologically valid, it lacks performance aspect *in situ*. Thus, the application in a real-life setting of EF is not observed and assessed.

This review included BADS-C, a performance-based measure of EF intended for individuals aged 7-16 years old and contains tasks that measure EF used in specific tasks reflecting daily life activities [28]. BADS-C development was not within the occupational therapy context; however, the referred validation study was conducted concerning occupational therapy [20]. BADS-C has been reported in the occupational therapy literature, despite its grounding on neurophysiological perspectives, reflecting the utility of performance-based EF tools across professions. The reviewed performance-based EF tools reported in this study (while developed, tested, or used in the occupational therapy profession) may have implications on its use across other disciplines whose concern is children's EF.

This review found low certainty of evidence on the measurement properties of BADS-C, CCT, CKTA, Do-Eat, and PETA as EF instruments for children in OT practice. The available yet limited evidence supporting these tools contribute to the decision suggesting that their true measurement property effects might be markedly different from the estimated effect. The low rating in evidence certainty is due to various limitations commonly in the number of available studies reviewed, study quality, inconsistency of measurement properties, and probability of reporting bias. However, considering the critical need for evaluating EF among children [8] and the determinants of the strength of recommendations (GRADE [38]), the authors conditionally suggest using any of these EF tools. Nevertheless, future researchers will need to address further the adequacy of measurement properties of these EF tools.

Individual differences in EF have been seen in various childhood populations. EF can vary between children from different environments [39]. Developmental trajectories related to EF processes have likewise been suggested [40]. Thus, researchers will need to include contextualization of these EF instruments in different cultures, ages, and clinical populations in the future.

### 5.1. Limitations

There are several limitations to this review. While we used the COSMIN criteria for adequacy of measurement properties and the Terwee criteria for synthesis for best evidence criteria, there are times when these criteria are difficult to interpret. Some ratings were based on the review authors' judgment and may have violated the standards, making the replication of this review challenging. The use of these criteria in assessing the methodological rigor of the included studies allowed us to appraise and assess these studies robustly. Second, the COSMIN framework is intended for patient-reported outcome measures, and there have been adjustments in its use for performance-based clinical assessment methods. Nevertheless, the methods reported in this review may provide a basis for future studies of the same nature. An extension of COSMIN to cover performance-based assessments will be a welcome development. Lastly, the limited number of studies reviewed may be from the stringent conceptualization of our inclusion criteria, specifically in deciding that the EF assessment tools must have been relevant to OTs. Our review may likely underestimate the breadth of the evidence on EF assessments for children; however, it provides a niche market for our intended end-users. Future research may need to review other EF assessment methods used as an adjunct to occupational therapy.

## 6. Conclusions

In the current review, we identified five performance-based EF instruments for children in the practice of OT: BADS-C, CCT, CKTP, Do-Eat, and PETA. These five tools assess a child's EF in real-life settings and age-appropriate activities within the context of their occupations. However, their adequacy of measurement properties is lacking, which led to low certainty in their evidence. Nevertheless, the authors conditionally suggest their use based on the critical need to measure children's EF. Future research will need to adequately address and report a complete set of measurement properties tested in different childhood populations across ethnicity, age groups, and clinical conditions.

## Figures and Tables

**Table 1:** Summary of included studies.

Author/s	Year	Instrument	Age	Population characteristics	Country of development/testing and language	Type of assessment	Executive function assessed	Tasks	Scoring
Engel-Yeger et al.	2009	Behavioural Assessment of the Dysexecutive Syndrome for Children	8-15 years	*n* = 208 TD children	Israel (Hebrew)	Performance-based assessment and a 20-item questionnaire for caregivers (Dysexecutive Questionnaire for Children, DEX–C)	Inflexibility, perseverance, novel problem solving, impulsivity, planning, and the ability to utilise feedback to moderate behaviour	Six subtests: (1) Playing card test (2) Water test (3) Key search test (4) Zoo map test 1 (5) Zoo map test 2 (6) Six-part test	Each subtest will have its own scoring guideline. However, generally, scores are derived from the number of tasks completed correctly and rules broken or errors
Chevignard et al.	2009	Children's cooking task	9-14 years	*n* = 28 (TBI = 10, TD = 18)	France (French)	Performance-based assessment	EFs assessed were not explicitly mentioned. However, the execution error assessed by the tool is related to volition, planning, goal direction, or task monitoring	The task entails preparing a chocolate cake and a fruit cocktail while following a recipe with the necessary ingredients and utensils on a table.	Scoring is based on a classification and quantification of errors and the qualitative analysis of the task.
Chevignard et al.	2010	Children's cooking task	8-20 years	*n* =46 (TBI =25, TD =21)	Australia (English)				
Fogel et al.	2020	Children's cooking task	10-14 years	*n* =81 (EFD =41, TD =40)	Israel (Hebrew)				
Rocke et al.	2008	Children's Kitchen Task Assessment	8-12 years	*n* = 49 TD children (African American; M = 10.4 ± 1.12 years)	USA (English)	Performance-based assessment	Initiation, organization, planning and sequencing, judgment and safety, and completion	The child is asked to make a playdough.	Scoring is based on the cues given. The type of cue given is scored from 0 to 5: 0 = no cues 1 = general verbal guidance 2 = gesture guidance 3 = direct verbal assistance 4 = physical assistance 5 = doing for the participant
Josman et al.	2010	Do-eat	5-6.5 years	*n* = 59 children (DCD = 30, TD = 29)	Israel (Hebrew)	Performance-based assessment	Attention, initiation, sequencing, transition from one activity to another, spatial and temporal organization, inhibition, problem solving, and remembering instructions	The child is asked to perform three tasks: (1) Make a sandwich (2) Prepare chocolate milk (3) Fill out a certificate of their outstanding performance	Test scores range from 1 (unsatisfactory performance) to 5 (very good performance).
Rosenblum et al.	2015	Do-eat	6-9 years	*n* = 47 (ADHD =23, TD =24)	Israel (Hebrew)	Performance-based assessment			
Downes et al.	2018	Preschool executive Task assessment	3-6 years	*n* = 166	UK (English)	Performance-based assessment	Working memory, distractibility, organization, emotional control	The task involves using an “ingredients” box with preprepared materials, a recipe book, a timer, and cueing/scoring sheets. The child follows a picture recipe book step-by-step, using the supplied materials, to make the final picture.	Scoring is based on a classification and quantification of errors and the qualitative analysis of the task.

Note: ADHD: attention-deficit hyperactivity disorder; DCD: developmental coordination disorder; EF: executive function; EFD: executive function deficits; OT: occupational therapist; TD: typically developing; TBI: traumatic brain injury.

**Table 2:** Summary of EF tools' measurement properties.

Instrument	Author	Year	COSMIN adequacy of measurement properties						
			Content validity	Structural validity	Internal consistency	Reliability	Construct validity	Cross-cultural validity	Criterion validity
BADS-C	Engel-Yeger et al.	2009					Age: significant differences exist between the different age groups between three age groups on the following: playing card test (*p* ≤ 0.001), water test (*p* ≤ 0.001), key search test (*p* ≤ 0.001), and zoo map test (*p* ≤ 0.001). Gender: not significant. Socioeconomic status: not significant. Parent's education: not significant.	Underwent forward (Hebrew) and backward (English) translations by a bilingual clinician.	
CCT	Chevignard et al.	2009				Interrater reliability Total number of errors: ICC = 0.96 Types of errors: ICC = 0.70–0.99 Substitution sequence errors: ICC = 0.37	Group differences: significant differences exist only between the total number of errors (*p* ≤ 0.001), number of errors of each type (*p* ≤ 0.000‐0.007), and results of the qualitative analysis (*p* = 0.01‐0.12) of the cooking task in the TBI and control groups.		No significant correlation was found between the total number of errors in the cooking task and the scores on the different neuropsychological tests or behavioural questionnaires (i.e., RCF, WCST, TMT-B, Tower of London, six-part test, RBMT, BRIEF, DEX-C).
	Chevignard et al.	2010			Cronbach's *α* = 0.86	Test-retest reliability Total number of errors: ICC = 0.89 Duration of the task: ICC = 0.94 Types of error: ICC = 0.75‐0.90 Substitution-inversion: ICC = 0.68 Estimation errors: ICC = 0.46 Purposeless action: ICC = 0.59	Age: the total number of errors in the CCT significantly decreased with age in the control group (rs = −0.454; *p* ≤ 0.04) and in the TBI group (rs = −0.552; *p* = 0.004). Group: there is significant difference only in the total number of errors between TD and TBI children (*p* ≤ 0.01).	The CCT was translated. In English, recipes in the cookbook and the utensils were mildly changed, as quantities were expressed in “cups” and “tablespoons” instead of glasses. The CCT was trialled by one examiner and three typically developing children, indicating that instructions and recipes were clear and understandable.	Overall, performance in the CCT was significantly correlated (*p* ≤ 0.05) to general cognitive ability, to some of the cognitive tests of executive functions on the D-KEFS (trails, verbal fluency, sorting, twenty questions), and the cognitive subscale of the DEX-C questionnaire.
	Fogel et al.	2020					Group: significant differences were found between the groups in the CCT assessment scores (*p* ≤ 0.010‐0.001). Error types: discriminate function was found for group classification of participants in the descriptive (*p* ≤ 0.001) and neuropsychological (*p* ≤ 0.001) analyses of the CCT.	The CCT was translated into Hebrew through a process of forward and backward translations. Content validity was pilot tested on a group of five children and a focus group of seven OTs.	A medium positive correlation was found only between the BRIEF-SR subscales plan/-organization (*r* = 0.31, *p* ≤ 0.05) and task duration.
CKTA	Rocke et al.	2008			Cronbach's *α* = 0.68	Interrater reliability: ICC = 0.98	Performance significantly improved as age increased (ns). Can discriminate between high- and low-scoring participants when compared to the BRIEF (inhibition: *p* ≤ 0.003, BRI: *p* ≤ 0.01), D-KEFS Confirmed Correct Card Sorts (*p* < 0.03), and WISC-IV Digit Span backwards (*p* < 0.04).		
Do-eat	Josman et al.	2010	Content and face validity: validated by five expert consultants and five experienced pediatric occupational therapists.		Cronbach's *α* = 0.89	Interrater reliability: ICC = 0.92‐1.00	Construct validity for the Do-Eat was assessed by gauging the tool's ability to distinguish between the groups of children with and without DCD and found significant differences in executive functions (*p* ≤ 0.001).		The EF task was not specifically correlated to any EF assessment.
	Rosenblum et al.	2015			Cronbach's *α* = 0.877		Significant group differences were found in the EF scores (*p* ≤ 0.001) with and without ADHD.		Significant correlations were found in the ADHD group between the EF Do-Eat score for “preparing chocolate milk” and BRIEF BRI (*p* ≤ 0.05) and MI (*p* ≤ 0.05) scores only.
PETA	Downes et al.	2018				Interrater reliability: ICC = 0.93 Intrarater reliability: ICC = 0.88‐0.98	Age: performance significantly increased with age in line with the rapid development of executive skills reported during this period (*p* ≤ 0.001). Chronological age predicted 40% of the variance in TS (*p* ≤ 0.001). Age was strongly related to performance on all quantitative domains of the PETA (TS, TC, initiation, sequencing, metacognition, completion, time for completion; *p* ≤ 0.05), except for judgment/safety. Domain scores: examiner ratings of organization during the PETA task showed that the poor PETA group obtained the poorest teacher ratings on the BRIEF-P plan/organize domain, followed by the typical group and the very good group (*p* = 0.05). Other results were not significant.		The PETA TS was compared with the BRIEF-P GEC. A significant association was observed between the PETA TS and the BRIEF-P GEC (*p* ≤ 0.001). Other correlations were not significant.

Note: EF: executive function; BADS-C: Behavioural Assessment of the Dysexecutive Syndrome for Children; CKTA: Children's Kitchen Task Assessment; DEX-C: Dysexecutive Syndrome for Children; PETA: Preschool Executive Task Assessment; TS: total summary score; TC: total number of cues; ICC: intraclass correlation; GEC: general executive composite; BRIEF: Behavior Rating Index of Executive Function; BRIEF-SR: Behavior Rating Index of Executive Function-Self-Report; BRIEF-P: Behavior Rating Index of Executive Function-Preschool; BRI: Behavioural Regulation Index; MI: metacognition index; RBMT: Rivermead Behavioural Memory Test; RCF: Rey-Osterrieth Complex Figure; D-KEFS: Delis–Kaplan Executive Function System; WCST: Wisconsin Card Sorting Test; WISC-IV: Wechsler Intelligence Scale for Children-IV; TMT-B: Trail Making Test Part B; DCD: developmental coordination disorder; ADHD: attention-deficit hyperactivity disorder. Empty cells: no evidence found.

**Table 3:** Summary of adequacy of EF tools' measurement properties.

Instrument	Author	Year	COSMIN adequacy of measurement properties						
			Content validity	Structural validity	Internal consistency	Reliability	Construct validity	Cross-cultural validity	Criterion validity
BADS-C	Engel-Yeger et al.	2009					+	?	
CCT	Chevignard et al.	2009				+	−		−
CCT	Chevignard et al.	2010			+	+	−	?	−
CCT	Fogel et al.	2020					+	?	−
CKTA	Rocke et al.	2008			−	+	−		
Do-Eat	Josman et al.	2010	?		+	+	+		−
Do-Eat	Rosenblum et al.	2015			+		+		−
PETA	Downes et al.	2018				+	−		−

Note: BADS-C: Behavioural Assessment of the Dysexecutive Syndrome for Children; CCT: Children's Cooking Task; CKTA: Children's Kitchen Task Assessment; PETA: Preschool Executive Task Assessment. Legends (**−**, ?, **+**) are explained in Supplementary File 2. Empty cells: no evidence found.

**Table 4:** Summary of synthesis of best evidence and GRADE rating on evidence certainty for the reviewed EF tools.

Measure	Synthesis of best evidence							GRADE rating on certainty of evidence
	Content validity	Structural validity	Internal consistency	Reliability	Construct validity	Cross-cultural validity	Criterion validity	
BADS-C					+++	+		Low
CCT			+	+	±	++	±	Low
CKTA			+	+	+			Low
Do-eat	+		+++	++	+++		±	Low
PETA				+++	++		±	Low

Note: BADS-C: Behavioural Assessment of the Dysexecutive Syndrome for Children; CCT: Children's Cooking Task; CKTA: Children's Kitchen Task Assessment; PETA: Preschool Executive Task Assessment. Legends used for synthesis of best evidence (**±**, **+**, **++**, **+++**) are explained in Supplementary File 3, while the GRADE rating (i.e., low) is explained in Supplementary File 4. Empty cells: no evidence found.

## Data Availability

All pertinent data related to the reported systematic review has been included in this article. Inquiry on other data may be requested from the primary author.

## Supplementary Materials

Supplementary 1 search strategy.

Supplementary 2 COSMIN criteria for adequacy of measurement properties.

Supplementary 3 synthesis of best evidence criteria.

Supplementary 4 GRADE rating certainty in the evidence.
