# Supplementary material for: Performance-Based Executive Function Instruments Used by Occupational Therapists for Children: A Systematic Review of Measurement Properties
Source: Occup Ther Int. 2021 Aug 6;2021:6008442. doi: 10.1155/2021/6008442 (PMC8374859; doi:10.1155/2021/6008442)
Supplement: Supplementary 1 — search strategy. [file 6008442.f1.docx]

**Supplementary File 1. Search strategy**

| Keyword | Keyword alternatives |
| --- | --- |
| Executive functions | Executive+function* *OR* “EF” *OR* executive *OR* “central executive” |
| Children | Child* *OR* school-age *OR* kids *OR* youth |
| Occupational therapy | “OT” *OR* “occupational therapist” |
