# Supplementary material for: Performance-Based Executive Function Instruments Used by Occupational Therapists for Children: A Systematic Review of Measurement Properties
Source: Occup Ther Int. 2021 Aug 6;2021:6008442. doi: 10.1155/2021/6008442 (PMC8374859; doi:10.1155/2021/6008442)
Supplement: Supplementary 2 — COSMIN criteria for adequacy of measurement properties. [file 6008442.f2.docx]

**Supplementary File 2. COSMIN criteria for adequacy of measurement properties**

| **Measurement Property** | **Rating** | **Criteria** |
| --- | --- | --- |
| Content validity (including face validity) | + | All items refer to relevant aspects of the construct to be measured AND are relevant for the target population AND are relevant for the purpose of the measurement instrument AND together comprehensively reflect the construct to be measured |
|  | ? | Not all information for ‘+’ reported |
|  | - | Criteria for ‘+’ not met |
| Structural validity | + | Unidimensionality: EFA: First factor accounts for at least 20% of the variability AND ratio of the variance explained by the first to the second factor >4 OR Bi-factor model: Standardized loadings on a common factor > 0.30 AND correlation between individual scores under a bi-factor and unidimensional model >0.90 Structural validity: CFI or TLI or comparable measure >0.95 AND (Root Mean Square Error of Approximation (RMSEA) <0.06 OR Standardized Root Mean Residuals (SRMR) < 0.08) |
|  | ? | Not all information for ‘+’ reported |
|  | - | Criteria for ‘+’ not met |
| Internal consistency | + | At least limited evidence for unidimensionality or positive structural validity AND Cronbach's alpha(s) ≥0.70 and ≤0.95 |
|  | ? | Not all information for ‘+’ reported OR conflicting evidence for unidimensionality or structural validity OR evidence for lack of unidimensionality or negative structural validity |
|  | - | Criteria for ‘+’ not met |
| Reliability (Internal and Test-Retest) | + | ICC or weighted Kappa ≥0.70 |
|  | ? | ICC or weighted Kappa not reported |
|  | - | Criteria for ‘+’ not met |
| Construct validity (hypothesis testing) | + | At least 75% of the results are in accordance with the hypotheses |
|  | ? | No correlations with instrument(s) measuring related construct(s) AND no differences between relevant groups reported |
|  | - | Criteria for ‘+’ not met |
| Cross-cultural validity | + | No important differences found between language versions in multiple group factor analysis or DIF analysis |
|  | ? | Multiple group factor analysis AND DIF analysis not performed |
|  | - | One or more criteria for ‘+’ not met |
| Criterion validity | + | Convincing arguments that gold standard is “gold” AND correlation with gold standard ≥0.70 |
|  | ? | Not all information for ‘+’ reported |
|  | - | Criteria for ‘+’ not met |
