# Supplementary material for: Performance-Based Executive Function Instruments Used by Occupational Therapists for Children: A Systematic Review of Measurement Properties
Source: Occup Ther Int. 2021 Aug 6;2021:6008442. doi: 10.1155/2021/6008442 (PMC8374859; doi:10.1155/2021/6008442)
Supplement: Supplementary 3 — synthesis of best evidence criteria. [file 6008442.f3.docx]

**Supplementary File 3. Synthesis of best evidence criteria**

| **Level** | **Rating** | **Criteria** |
| --- | --- | --- |
| Strong | +++ or --- (total sample size > 100) | Consistent findings in multiple studies of good methodological quality or one study of excellent methodological quality |
| Moderate | ++ or -- (total sample size 50–99) | Consistent findings in multiple studies of fair methodological quality or in one study of good methodological quality |
| Limited | + or - (total sample size 25–49) | One study of fair quality |
| Conflicting | ± | Conflicting findings |
| Unknown | ? (total sample < 25) | Only studies of poor methodological quality |
