# Supplementary material for: Performance-Based Executive Function Instruments Used by Occupational Therapists for Children: A Systematic Review of Measurement Properties
Source: Occup Ther Int. 2021 Aug 6;2021:6008442. doi: 10.1155/2021/6008442 (PMC8374859; doi:10.1155/2021/6008442)
Supplement: Supplementary 4 — GRADE rating certainty in the evidence. [file 6008442.f4.docx]

**Supplementary File 4. GRADE rating certainty in the evidence**

| **Certainty** | **Meaning** |
| --- | --- |
| Very Low | The true effect is probably markedly different from the estimated effect |
| Low | The true effect might be markedly different from the estimated effect |
| Moderate | The authors believe that the true effect is probably close to the estimated effect |
| High | The authors have a lot of confidence that the true effect is similar to the estimated effect |
